# Domestic Hygiene

**Published:** 1881-09

**Authors:** 


### POPULAR SCIENCE DEPARTMENT.

### PROFESSOR FRANK DONALDSON, M. D., ON DOMESTIC HYGIENE.

(Continued from last issue.)

*High Temperature.*—A prominent cause of the unhealthfulness of our houses is the high temperature at which they are kept. Day and night the thermometer frequently registers 75 degrees, or even higher, when 65 degrees ought to be the highest for day hours, unless for very old people or the enfeebled, and 60 degrees the maximum of the night hours. Yet many think the room unbearable if the mercury stands below 72 degrees or even 75 degrees. During sleep 55 degrees or 58 degrees in winter is the best.

*Every unnecessary degree of artificial heat lessens our power to generate heat for ourselves.* Our animal temperature, it must be borne in mind, is the result of the activities, the movements, and the formations and transformations of our bodies; in one word, it is the result of the functions of our bodies, heat being nothing more nor less than a form of motion inside as outside of the body. If the temperature of our rooms be kept at the degree proper for health, the heat-producing powers of the body will remain vigorous, and consequently the skin, the evaporation of its perspiration keeping down the animal temperature, will be kept at a comfortable degree of warmth. In over-heated air the natural powers of the body become indolent, and therefore produce less heat. (Osgood.)

The result is loss of bodily power; for the more heat the less vigor. As Dr. Osgood says with force: "We should train our sensations and watch them, so that when the mercury begins to creep up above its proper place we may notice the change, and at once take means to lessen the heat of the room, otherwise a person never knows whether the temperature be correct."

He begins the day in a room at 68 degrees. In a few hours it has reached 75 degrees or 80 degrees. Heat is an extremely seductive thing. Immoderate degree of heat during the winter is the cause of much illness—a daintily housed person has no power to resist cold.

In our variable climate we cannot escape sudden changes from warm to cold air. If our bodies are properly clothed and we are accustomed in our homes to low temperature, we can meet the transitions with comparative freedom from danger of chill. Some one has said that "our body, debilitated by overheat in rooms, is like a figure of wax; it droops, loses its firmness, and becomes excessively relaxed. No part of the body is as sensitive to heat as the skin. We know how freely it acts in hot weather and in hot rooms."

When fresh air is shut out, the skin becomes less and less able to resist changes of temperature. When the individual goes out, no matter how much clothing he may wear, he suffers, and a cold, a rheumatism or perhaps a pneumonia is the result. The cold air inhaled into the lungs, and the chilled and chilling blood from the skin rushes into the internal organs. Fortunately only a portion of the blood passes through the skin. If it did, sudden death and dangerous lung and throat affections, which, as it is, cost more lives than do almost all other diseases together, would be still more frequent in our rigorous winters. Lack of power to resist cold is a forerunner of evils which develop into those tiresome and hard-to-cure catarrhal affections so common in our climate. High temperatures depress our *mental powers*. No student, no thinker can possibly work efficiently when the hot air prevents a healthful circulation with the brain. (Osgood.) We feel weary, sleepy, incapable of work; the brain as well as the lungs, the heart, and other vital organs, become inactive. But the greatest sufferers from over-heated houses are the helpless children, who frequently fall victims to the lamentable ignorance of their mothers of the simplest principles of hygiene. They are literally smothered by being forced to breathe devitalized air, and their feeble organisms are exhausted by its high temperature. It saddens one to reflect upon the fearful mortality among the young children in our cities. Nearly *one-third die in their first year*, *one-half before the end of their fifth year*. Vital statistics prove that this results, in a great degree, from preventible causes, and that foul air and excessive heat contribute to it. They are made to suffer for the hygienic sins of their careless parents.

The adult judges by his sensations, but the children do not understand or appreciate theirs; they are not sedentary; they are active, ever on the go, and therefore manufacture heat for themselves—all they need is a degree of heat which will not too rapidly deprive them of their warmth. The thermometer rises among the seventies and eighties, the little ones continuing to play with their usual vigor; they at once perspire freely and soon become exhausted; they become excessively sensitive to the slightest chill or change of air; they become restless and fretful; they soon become wakeful, pale, inactive, no appetite for their food, croups, pneumonia, &c., &c.

An enfeebled child is in constant danger. The physicians preach and complain, but the benighted parents only think of one point, the danger of their children catching colds, when they, in reality, by over-heat, cause them to do so.

One other effect of over-heated air should be mentioned—the great dryness it creates.

In winter the air is usually very dry. Upon being heated it has an immense affinity for moisture, and as Osgood and Leidy say, if this moisture be not then supplied in some other way, it will take the natural humidity from the skin and lungs, thus creating the dry, parched, feverish condition so noticeable in rooms heated by stoves and furnaces. *The higher the temperature the greater should be the quantity of moisture supplied.* For every increase of 27 degrees temperature air doubles its capacity for holding water. Too much heat is bad enough, but if it be also dry the danger to the throat, heart and lungs is very great.

The head of the house should see that the evaporating pans are kept supplied with water. Every stove should have its pan of water. Each and every register through which the heat comes should have its reservoir of water. Even when open fires are used there should be water evaporated. The sextons of churches and the proprietors of public halls and theatres by their neglect in not furnishing water for evaporation, cause the public much discomfort, and frequently positive disease. It must not be forgotten that the very young, inactive infants as well as the very aged require higher temperature than others.

But the air must be purified and changed by ventilation more frequently than when at a low temperature.

*Warming Houses.*—The point to be especially aimed at in warming houses is not only to keep the temperature up to the proper degree, but to see that the warmed air of the room is changed *often enough to keep it pure*, and that the normal moisture is retained—

ventilation and heating must go together. For health, unquestionably the primitive blazing wood-fire has everything in its favor. It is pleasant and cheerful, and keeps the air fresh. The inmates of the rooms have to change their positions in order to keep all sides warm. But in modern houses, furnaces and fire-place coal stoves have to be resorted to. There are serious hygienic objections to their use, yet, if properly managed and regulated, these objections may be obviated. Valves and dampers used carefully and skillfully will obviate them in a great measure. If it is remembered that they furnish warm air to *enter* the rooms, but that of itself, it affords no *outlet* for its escape or for the escape of used up air, if provision is made for an *outlet* and for *supplying moisture*, they may not prove injurious. They save so much trouble and prevent the accumulation of dirt. Care must be taken that heat be supplied to the halls—otherwise the cool air will rush in when the doors are left open, and there will be marked difference, perhaps of 12 degrees or 20 degrees between the floor and the height to which a man can reach. This is the reverse of what it ought to be, since to *keep the head cool and the feet warm*, is an *old* and approved maxim of health. One disadvantage of furnace heat is that it requires for the comfort of the inmates a higher temperatrue than if an open fire of wood or coal is used. Workshops, skating rinks, and gymnasiums should not be kept over 55 degrees. Sitting rooms and lecture rooms should be kept warmer—never over 65 degrees for young people.

Permit me to suggest some simple rules for the regulation of furnaces. "They should be large in proportion to the house, so that a moderate fire, not *pushed*, may supply enough warm air. A very hot fire *bakes* the air, causing an unpleasant odor and an uncomfortable feeling through the house. If the furnace be too small, it will not afford enough heat without driving the fire hard in cold weather. Air should be supplied to the air-chamber directly from out of doors, because cellar air cannot be as pure—indeed, it frequently contains many impurities."—*Hartshorne.*

Again, water should always be placed in the air-chamber to be evaporated and thus prevent undue dryness, for a certain amount of moisture in the atmosphere is essential to life itself. Every one is familiar with the destructive effects of the Simoon and Harmatton winds of Arabia and Africa, which carry the scorching aridity of the desert with them, blasting everything that has life. Warming the air increases its capacity for retaining moisture without saturation. By raising the temperature of the air in any apartment from 50 degrees to 70 degrees, the relative humidity may be reduced from 100 degrees to 25 degrees ; that is, from containing moisture enough for saturation down to one-quarter of that amount ; of course, then, a sufficient quantity of vapor must be added to bring it up to twice as much or more, say to 68 degrees.

How seldom this is done we all know from the oppression we feel in over-heated houses.

Great care should be taken that no leakage of gas occurs from any flue or pipe into the air-chamber. Coal gas is poisonous when concentrated. Death has been produced by its insidious entrance into the air of occupied rooms.

It produces headache and discomfort attributed to other causes.

Warm air should be supplied to halls so as to make no difference in temperature when doors are open.

Since no outlet belongs to the furnace system, the needful arrangement must be supplied. Nothing can equal a "low-down" grate or a small open wood fire. Some plan for letting the air out by Arnott's or Barker's flues, or an open window crack, so arranged as not to produce a draught. *One cardinal rule must be insisted upon, and that is that the fire-places shall never be closed.* A small piece of wood or some small amount of coal will carry the air of the room to the top of the house.

